# Genome-wide phylogeny reshapes our understanding of the evolution of deep-sea dragonfishes, bristlemouths, viperfishes, and allies (Stomiiformes)

**DOI:** 10.1186/s12862-025-02453-0

**Published:** 2025-10-21

**Authors:** Solomon Chang, Zach Heiple, Delson Hays, Fernando Melendez-Vazquez, Casey Lee, Benjamin W. Frable, John Pogonoski, Christopher M. Martinez, Ricardo Betancur-R, Dahiana Arcila

**Affiliations:** 1 Scripps Institution of Oceanography, University of California San Diego, 9500 Gilman Drive, La Jolla, CA 92093-0244 USA; 2 CSIRO Australian National Fish Collection, National Research Collections Australia, Hobart, TAS Australia; 3 University of California, Irvine, Irvine, CA 92697-2650 USA

**Keywords:** Mesopelagic Fishes, Taxonomic Classification, Morphological Diversity

## Abstract

**Background:**

The evolutionary relationships within Stomiiformes, a diverse order of deep-sea fishes dominating the mesopelagic and bathypelagic zones, remain contentious due to conflicting morphological and molecular evidence. These fishes, comprising 464 species across four traditionally recognized families (Gonostomatidae, Sternoptychidae, Phosichthyidae, and Stomiidae), exhibit remarkable adaptations such as bioluminescence, ultra-black pigmentation, and extreme jaw morphologies. Their global abundance and ecological significance, including contributions to the biological carbon pump, underscores the need to resolve their phylogeny amid escalating threats from climate change and human activities.

**Results:**

We conducted the most comprehensive phylogenomic analysis of Stomiiformes to date, integrating 936 nuclear loci from 60 species and an expanded dataset of 135 species with mitochondrial sequences from publicly available repositories such as the Barcode of Life Data Systems (BOLD) database. We used maximum likelihood and coalescent-based approaches to assess family monophyly and relationships, including extensive quality control to address contamination in public databases. Our analyses reveal unstable tree topologies and complex evolutionary histories that challenge traditional classifications, while our quality control analyses identified 29% of BOLD sequences as misidentified or contaminated, emphasizing rigorous curation for deep-sea taxa. Congruent with a recent taxonomic treatment of Stomiiformes, the families Phosichthyidae and Gonostomatidae exhibit polyphyly and paraphyly, respectively, while subfamilies within Stomiidae are extensively non-monophyletic, leading us to recommend their abandonment. We propose the recognition of eight monophyletic families: Vinciguerriidae, Diplophidae, Gonostomatidae, Yarrellidae, Ichthyococcidae, Phosichthyidae, Sternoptychidae, and Stomiidae, supported by robust molecular and morphological evidence.

**Conclusions:**

This revised classification reflects the morphological and ecological diversity of Stomiiformes, aligning with their evolutionary diversification in the deep sea. Our phylogenomic framework resolves longstanding systematic uncertainties and highlights the power of genome-wide data in tackling taxonomically challenging clades. These findings provide a foundation for understanding deep-sea fish diversification and assessing the potential ecological drivers for their evolutionary diversity.

**Supplementary Information:**

The online version contains supplementary material available at 10.1186/s12862-025-02453-0.

## Introduction

Despite remarkable progress in unraveling the diversity of life through phylogenomic approaches, the evolutionary relationships among major clades of deep-sea fishes remain a subject of intense debate and uncertainty. This uncertainty is particularly pronounced in groups that have undergone extensive phenotypic adaptations to deep-sea environments, raising questions about the reliability of traditional taxonomic groupings [1]. Addressing this fundamental challenge in deep-sea systematics is crucial for understanding how fishes evolve and diversify in Earth’s largest and least accessible habitat, particularly as ecosystems face unprecedented pressures from global warming and anthropogenic activities. Deep-sea fishes also play a vital role in the biological carbon pump, facilitating carbon transport from surface waters to the deep ocean through vertical migrations, metabolic processes, and trophic interactions [2]. Elucidating their evolutionary relationships is crucial to understanding how their unique adaptations have facilitated their dominance in meso- and bathy-pelagic habitats.

The order Stomiiformes, encompassing dragonfishes, lightfishes, loosejaws, viperfishes, bristlemouths, and hatchetfishes, stands out as one of the most successful and diverse groups of deep-sea fishes, with over 460 species across four traditionally recognized families [3]. These fishes dominate the mesopelagic and upper bathypelagic zones [4], exhibiting a striking array of morphological adaptations, such as elongated or compressed deep-bodied shapes [5, 6], transparent teeth [7], ultra-black pigmentation, bioluminescence, and extreme jaw morphologies [6–8]. Despite their ecological significance, the evolutionary relationships within Stomiiformes remain highly controversial, as traditional morphology-based classifications frequently conflict with molecular data [8–13].

**Fig. 1:**
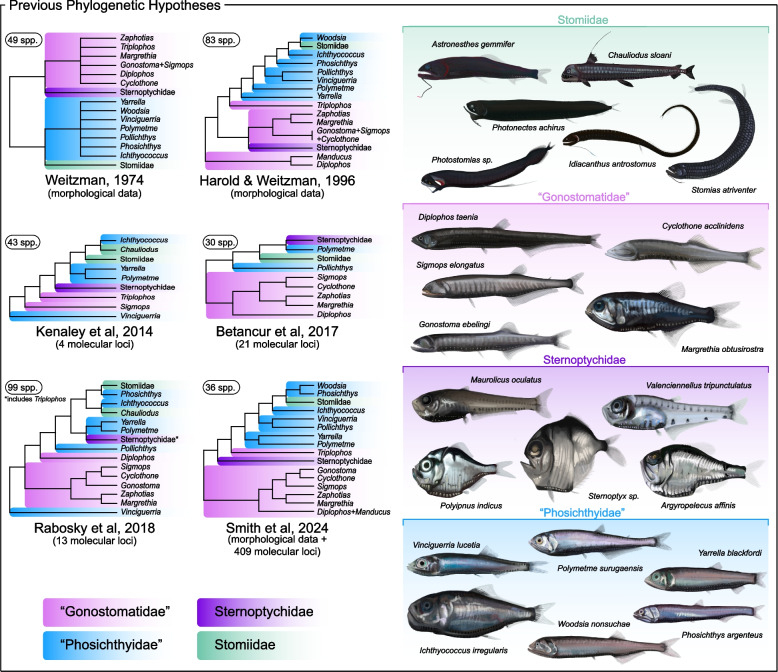
Previous phylogenetic hypotheses (left) and illustrations of select stomiiform species organized based on previous family-level taxonomy (right). This figure includes a conglomeration of both past morphological and molecular analyses of this order. The number of species (spp.) and markers are indicated when appropriate. Families are color-coded as Stomiidae (green), Sternoptychidae (purple), “Phosichthyidae” (blue), and “Gonostomatidae” (pink). The right panel provides accurate morphological illustration of several species from their respective families to highlight the morphological diversity within the Stomiiformes. Illustrations by Casey Lee

Previous phylogenetic studies have produced inconsistent results regarding family-level classifications and genus-level delimitations [14–18]. Early parsimony-based morphological analyses recognized as many as ten families, but subsequent studies consolidated these into four currently accepted families: Gonostomatidae, Sternoptychidae, Phosichthyidae, and Stomiidae [15, 17–19] (Fig. 1). These revisions expanded Stomiidae to include genera previously classified in their own families such as Astronesthidae, Chauliodontidae, Idiacanthidae, Malacosteidae, and Melanostomiidae, yet genus-level delimitations within Stomiidae remain poorly supported and lack resolution [8, 9, 12, 13, 20, 21]. Early molecular studies relying on mitochondrial markers largely corroborated morphology-based phylogenies for Gonostomatidae and Sternoptychidae, but offered limited insights into Stomiidae’s phylogenetic delineations [14, 19, 20, 22–24]. Later analyses incorporating nuclear loci revealed a more complex evolutionary history, demonstrating the polyphyly of Phosichthyidae, the paraphyly of Gonostomatidae, and sometimes the paraphyly of Stomiidae [8, 25]. Most recently, a study of 35 species, integrating ultraconserved elements, legacy markers, and morphological data, further reduced the number of recognized stomiiform families to three by subsuming all seven phosichthyid genera and one gonostomatid genus into Stomiidae [13].

Given the logistical challenges and high costs of collecting fresh tissue from deep-sea fishes, molecular studies of stomiiform relationships have been severely constrained by limited taxonomic and genetic sampling, with most analyses focusing on a small subset of species or specific families [20, 23, 24]. Although next-generation sequencing now offers powerful tools to resolve evolutionary relationships, these advanced methods have yet to be comprehensively applied to the order Stomiiformes. As a result, critical questions about this diverse order remain unresolved, including the validity of traditional family-level classifications, the phylogenetic placement of key genera, and the monophyly of major lineages. This gap in our understanding is particularly striking given the ecological importance of Stomiiformes in deep-sea ecosystems and their remarkable adaptations that lack the robust phylogenetic context necessary for evolutionary inquiry.

Here, we present the most comprehensive phylogenomic analysis of Stomiiformes to date, integrating multiple molecular datasets to resolve relationships across the order with unprecedented taxonomic breadth. Our approach combines a genome-wide dataset for 60 species, with an expanded dataset of 86 additional species, sourced from nearly 2,500 sequences in the BOLD database. Leveraging this extensive taxonomic sampling, we aim to 1) reconstruct higher-level evolutionary relationships within Stomiiformes, 2) test the monophyly of traditionally recognized families, and 3) evaluate previously proposed classifications at family and subfamily levels. Our study resolves longstanding systematic uncertainties, provides a robust phylogenetic framework for the order, and establishes a revised classification based on both molecular and morphological evidence. We also identified frequent instances of misidentification or contamination in stomiiform mtDNA sequences within the BOLD database, underscoring the need for cautious interpretation of such data. This finding emphasizes the critical role of rigorous data curation, particularly for deep-sea taxa where sampling challenges and identification problems are pronounced. Ultimately, our study highlights the power of integrating new genomic data with existing sequence resources to resolve relationships in taxonomically challenging radiations.

## Results

### Phylogenomic analyses reveal novel deep-sea fish relationships

**Fig. 2:**
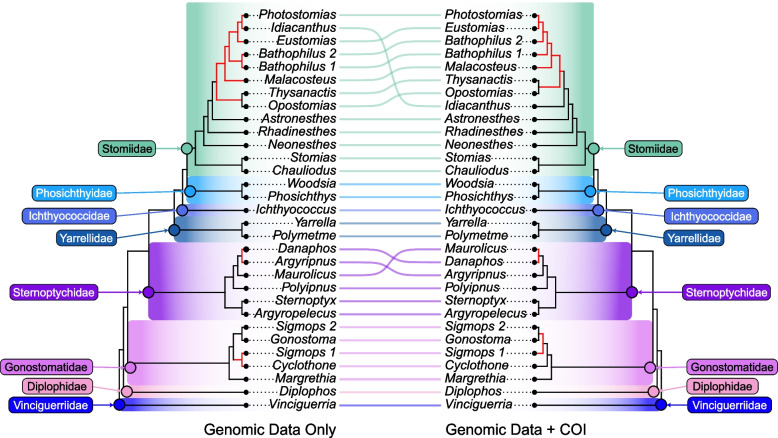
Tanglegram showing concatenation-based stomiiform topologies generated by genomic data only (left) and genomic data supplemented with COI sequences (right). Non-monophyletic genera (*Bathophilus* & *Sigmops*) represented as distinct clades (e.g. *Bathophilus* 1 and *Bathophilus* 2). Topological discordance indicated with red branches. Family-level classifications follow the new classification system proposed herein

Our primary nuclear exon-based phylogenomic dataset included 60 stomiiform species [31 genera, 4 families] and four outgroup taxa, comprising 936 loci totaling 314,607 base pairs (bp), with 73% missing data and a mean of 20 species per locus. To address potential biases from missing data and assess phylogenetic uncertainty, we applied stringent filtering strategies, generating a highly filtered dataset (37 loci: 10,872 bp, 49% missing data), two medium-filtered datasets (401 loci: 160,632 bp, 62% missing data; 472 loci: 223,738 bp, 71% missing sites), and three subset datasets (each 318 loci: 103,515–109,623 nucleotides with 72.14%−73.68% missing sites) (Additional file 1). Maximum likelihood (ML) analyses in IQ-TREE yielded congruent topologies across all datasets, with 78% of nodes in all our trees supported by bootstrap values (BS) > 85%. By contrast, coalescent-based species trees (ASTRAL-IV), inferred from individual gene trees, exhibited significantly lower support values, sharing only 54.4% of nodes on average with concatenated ML trees. Despite substantial discordance in branching patterns between coalescent- and concatenation-based trees, the monophyly of most taxonomic groups proposed herein was consistently resolved (Fig. S1). An exception was Sternoptychidae, which was resolved as paraphyletic in all the coalescent-based analyses, but strongly supported as monophyletic in all concatenation-based analyses (Fig. 2; Fig. S1). The phylogenetic incongruences observed in coalescent-based trees are likely due to the high levels of gene tree estimation error. Given the documented gene tree error and the robust topological consistency across our concatenation-based datasets, we selected the ML tree, inferred from the full 936-locus dataset, as our primary phylogenetic hypothesis. Trees estimated using ASTRAL-IV are summarized in Fig. S1.

### Quality control of COI sequences from BOLD

To assess the reliability of publicly available data for phylogenetic analyses, we assembled a COI dataset by mining 2,474 sequences from the BOLD database and integrating them with COI data from 51 of the 60 species in our primary genomic dataset, resulting in 2,525 individuals with sequence lengths ranging from 567 to 1,563 bp (see Methods). We evaluated sequence quality by inferring an ML tree, followed by visual inspection of the phylogeny for species clustering, topological accuracy, and sequence identification. Sequences were grouped into 181 species-level bins based on consistent clustering (Additional file 2). Comparison of BOLD species names with their bin assignments revealed that 728 sequences (28.8%) failed our quality control (QC) protocol (Fig. S2). Of these, 146 sequences could not be assigned to any bin and were flagged as ‘ID cannot be determined,’ 424 lacked specific genus or species designations (e.g., Stomiiformes sp., *Stomias* sp.), and 158 showed mismatched bin assignments, indicating misidentification (Fig. S3; Additional file 3). The distribution of QC failures across families revealed that 20.9% of sequences from Gonostomatidae (99/468), 37.1% of Phosichthyidae (130/350), 33.7% of Sternoptychidae (236/701), and 26.3% of Stomiidae (263/998) were problematic (Fig. S3). Overall, 29% of the BOLD sequences (717/2,474) failed our QC protocol. However, genus-level clustering remained largely robust, indicating that inherent challenges associated with identification in this order are concentrated at the species level. To enhance data reliability, we recommend combining automated filtering based on sequence quality metrics with manual verification through preliminary phylogenetic analyses before final tree reconstruction.

### Phylogenetic analysis of an expanded multi-locus dataset

To maximize taxonomic coverage, we generated an expanded dataset comprising 135 stomiiform species and four outgroup taxa. We integrated our 936 nuclear loci data matrix (314,607 bp, 73% missing sites) with the COI locus (1,746 bp post-alignment), totaling 316,353 bp with an average of 88% missing sites and a mean of 16 species per locus (Additional file 1). ML analyses based on our expanded dataset in IQ-TREE yielded topologies that retained all major clade relationships from our primary 60-species phylogeny (Fig. 2). As a result of the high levels of missing data, driven by COI-only taxa, and trimmed COI alignment, this reduced resolution at several nodes and required extensive quality control steps. For instance, bootstrap support for Gonostomatidae (as defined herein) decreased from 94% in the primary dataset to < 55% in the expanded dataset. Within Gonostomatidae, the genus *Cyclothone* exhibited significant phylogenetic uncertainty, resolving within the family in only five of ten ML replicates. In other instances, we resolved a peculiar sister relationship between the gonostomatid genus *Cyclothone* and the phosichthyid genus *Vinciguerria*, however, this was likely due to long-branch attraction and missing data. Consistent with previous analyses, several stomiid genera (e.g., *Astronesthes, Thysanactis, Photostomias,* and *Photonectes*) have also had unstable placements within Stomiidae. This instability, compounded by variations in taxon sampling and proportionately high levels of missing data, underscores the challenges of resolving phylogenetic relationships with taxonomically expanded datasets, particularly for problematic clades like *Cyclothone* and the more speciose stomiid genera, such as *Astronesthes* and *Photonectes*. Even so, the resolution of higher-level clades were well preserved across most of our analyses (Fig. S1), especially our primary phylogeny (Fig. 2).

### Impact of sequence contamination on phylogenetic reconstruction

Given the high rate of contamination identified in the COI dataset, we assessed its impact on phylogenetic inference by reconstructing an ML tree using the 1,797 sequences that passed QC, excluding the 728 problematic sequences (Fig. S3). Compared to the full COI dataset, the filtered dataset improved topological stability for Gonostomatidae, with *Cyclothone* consistently resolving within the family (BS = 82%) rather than grouping with *Vinciguerria* as observed in some unfiltered analyses. However, stomiid subfamilies, including Astronesthinae and Stomiinae, continued to exhibit variable support (BS values ranging from 55 to 75%), suggesting that contamination is only one of several factors affecting fine-scale resolution in deep-sea Stomiiformes. A reanalysis of family-level clustering with the filtered dataset also showed improved support for newly proposed families within Stomiiformes, though some inconsistencies persisted compared to past molecular hypotheses (Table 1; Additional file 3). These findings emphasize the critical role of quality control in enhancing phylogenetic accuracy.

**Table 1:**
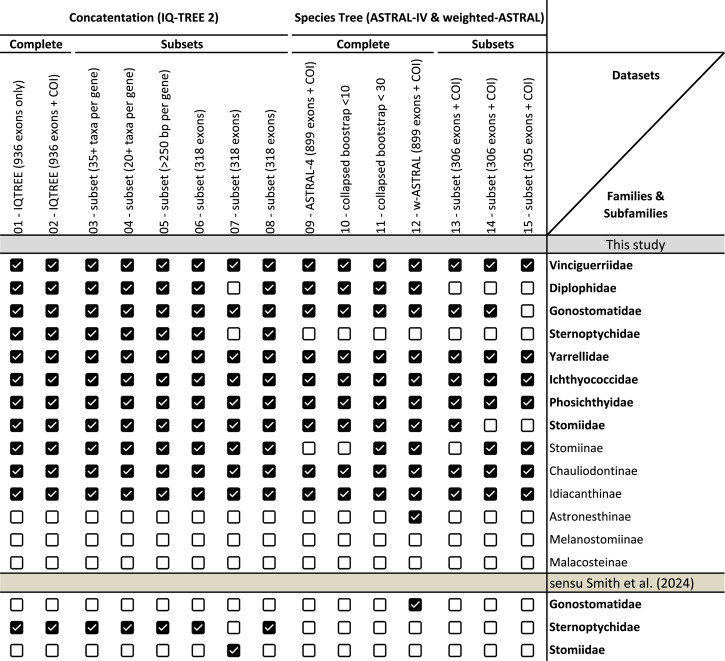
Assessment of family-level relationships within Stomiiformes using concatenation-based (IQ-TREE) and species tree (ASTRAL-IV & weighted-ASTRAL) methods across 15 datasets. Novel family-level clade comparison across different datasets using concatenation-based and multi-species coalescent methods. The right column includes all Stomiiform families as delimited in this study as well as Smith et al. [13]. Additionally, all previously acknowledged subfamilies of Stomiidae are included. Checkmarks indicate monophyly support for each family or subfamily. Families recognized herein are Vinciguerriidae, Diplophidae, Gonostomatidae, Sternoptychidae, Yarrellidae, Ichthyococcidae, Phosichthyidae, and Stomiidae, with previously-recognized Stomiidae subfamilies being: Stomiinae, Chauliodontinae, Idiacanthinae, Astronesthinae, Melanostomiinae, and Malacosteinae. Families sensu Smith et al. [13] are Gonostomatidae, Sternoptychidae, and Stomiidae

### Non-monophyly of traditional families reveals complex stomiiform relationships

**Fig. 3:**
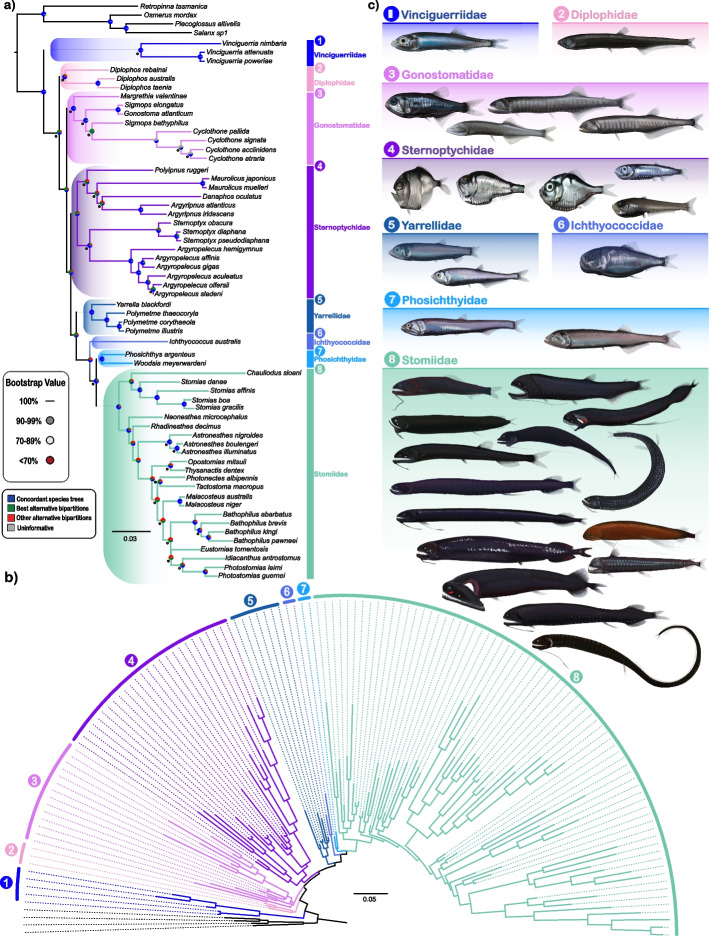
Family-level relationships and morphological diversity of Stomiiformes. **a** Molecular phylogeny derived from concatenation-based ML analysis of 936 exon markers, illustrating newly proposed family-level relationships. Monophyletic groups are color-coded by family. Nodes with bootstrap (BS) support < 100% are indicated by colored circles (90% and 70% thresholds), in addition to pie charts showing the proportion of concordant trees (blue), best alternative bipartitions (green), other alternative bipartitions (red), and uninformative trees (grey). All trees seen in Table 1 were included in the calculation of concordance of the 60 species phylogeny aside from the ASTRAL subsets. Exact BS values are provided in supplementary files. Numbers, color-coded by family, correspond to morphological illustrations in panel (**c**). **b** Radial representation of an expanded (135-taxon) phylogenetic tree based on COI + 936 exon markers, with branch lengths scaled to genetic divergence (scale bar = 0.05 substitutions per site). **c** Morphological illustrations of representative species from 31 genera across the families (1) Vinciguerriidae, (2) Diplophidae, (3) Gonostomatidae, (4) Sternoptychidae, (5) Yarrellidae, (6) Ichthyococcidae, (7) Phosichthyidae, and (8) Stomiidae, highlighting the morphological diversity within these groups. Families are consistently color-coded across all panels. Illustrations by Casey Lee

Our analyses fundamentally challenge traditional stomiiform taxonomy by revealing extensive paraphyly and polyphyly in two major families, Gonostomatidae and Phosichthyidae, respectively (as seen in Fig. 3a, b). The gonostomatids were resolved as two separate lineages, with *Diplophos* forming the sister group to all other taxa except the phosichthyid genus *Vinciguerria*, which diverges at the base of Stomiiformes (100% BS) (Fig. 3a). The remaining gonostomatid genera (*Cyclothone*, *Gonostoma*, *Margrethia*, *Sigmops*, *Triplophos*, and *Zaphotias*) (Fig. 3b) constitute a robustly-resolved Gonostomatidae sensu stricto, but its intrafamilial relationships are weakly supported and sensitive to changes in species sampling. Phosichthyidae is even more fragmented, with its seven genera scattered across four distinct lineages in nearly all the concatenation- and coalescent-based species trees (Table 1). Even after excluding the early-branching *Vinciguerria*, the remaining genera form three separate clades, rendering Phosichthyidae highly polyphyletic. One of the remaining three clades, hereafter referred to as Yarrellidae, unites *Polymetme* with *Yarrella* as the sister group to the other two remaining phosichthyid groups and the family Stomiidae. In our analyses, *Ichthyococcus* consistently emerged as an isolated lineage (hereafter, Ichthyococcidae) positioned between Yarrellidae and the newly defined Phosichthyidae (Table 2), now restricted to *Phosichthys* and *Woodsia* (Fig. 3a, b)*.* Of the phosichthyid clades, the sole genus *Vinciguerria* was substantially supported as sister to all other stomiiforms, and is relegated to the new family Vinciguerriidae (along with the morphologically-similar *Pollichthys*).

**Table 2:**
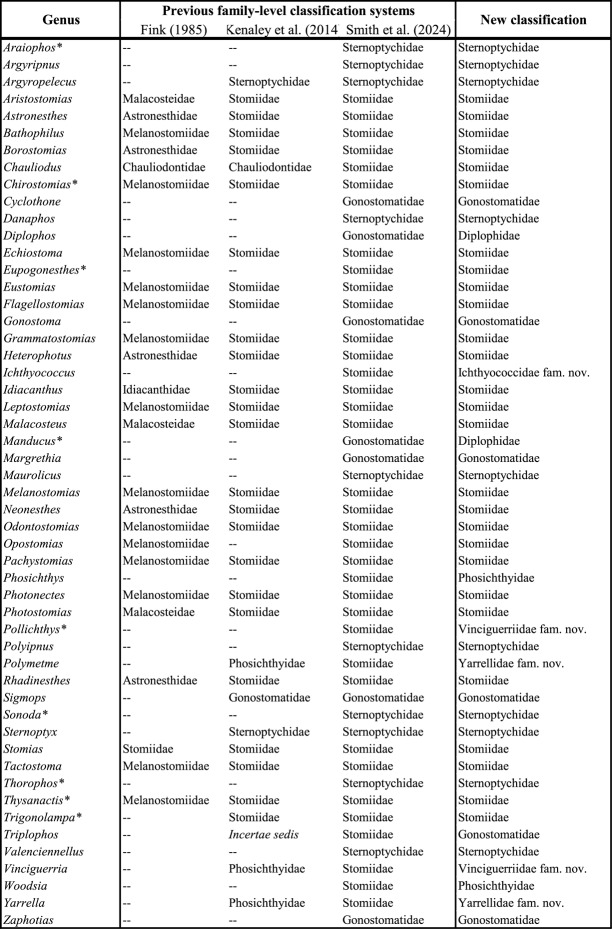
Systematic classification of Stomiiformes genera: traditional and newly proposed family-level groupings. Comparison of traditional family-level groupings from Fink (1985), Kenaley et al. (2014), and Smith et al. [13] with our newly proposed taxonomic arrangement based on phylogenomic evidence. Relevant stomiiform genera are denoted in the left column while their identified families based on different studies are listed coincidingly including this study. Our new taxonomic classification introduces novel families, including Ichthyococcidae fam. nov., Vinciguerriidae fam. nov., Yarrellidae fam. nov., and a newly revised Phosichthyidae and Gonostomatidae. Dashes (–) indicate missing genera from the study in question. Asterisks (*) highlight genera that have previously been placed in families such as Melanostomiidae, Malacosteidae, and Astronesthidae, which have now been reclassified based on modern phylogenomics.

### Stomiidae monophyly and Sternoptychidae conflict

Our analyses revealed robust support for a monophyletic Stomiidae (100% BS) (Fig. 3a, b) across all concatenated and most coalescent analyses (Table 1), offering new insights into the evolution of dragonfishes. This well-supported clade encompasses all six traditionally recognized subfamilies, exhibiting moderate to high levels of phylogenetic concordance with minimal conflicting bipartitions when comparing all our analyses to our most stable phylogeny, as indicated by the pie charts in Fig. 3a. However, their internal relationships within Stomiidae challenge existing classifications. Most notably, all three subfamilies containing multiple genera were resolved as polyphyletic (Malacosteinae and Melanostomiinae) or paraphyletic (Astronesthinae). Among these subfamilial groupings, Stomiinae was resolved as monophyletic (Table 1), most often forming a sister clade to a monophyletic Chauliodontinae. However, this latter relationship should be interpreted cautiously, as Chauliodontinae and Idiacanthinae were each represented by a single species in our primary dataset. Given the pervasive non-monophyly observed at both genus and subfamily levels in our analyses, consistent with findings from previous studies, we propose discontinuing the current subfamilial classification within Stomiidae. These artificial groupings appear to obscure, rather than clarify, our understanding of evolutionary relationships among dragonfishes, and their continued use may impede accurate systematic interpretation of this remarkable deep-sea radiation. The marine hatchetfishes (Sternoptychidae) showed strong support for monophyly in concatenated analyses (100% BS) (Fig. 3a) but yielded conflicting signals in species tree analyses (Table 1), which split the family in two distinct groups: *Sternoptyx* + *Argyropelecus* and *Argyripnus* + *Maurolicus* + *Polyipnus* + *Danaphos.* This similarly violates the traditional division of Sternoptychidae into two subfamilies (Sternoptychinae and Maurolicinae), and as such, we do not acknowledge the use of the sternoptychid subfamily classification. Additionally, the position of Sternoptychidae within Stomiiformes fluctuated across analyses (Table 1), indicating that increased taxonomic sampling may be necessary to resolve their phylogenetic position with greater confidence.

### Tree concordance factors reveal clade stability and conflict

To assess topological stability while avoiding the biases introduced by gene-tree error, we calculated tree concordance factors (tCFs), which measure the percentage of species trees that resolve each branching pattern in our main phylogeny. The tCFs was computed by using the 13 independently inferred phylogenies, including six concatenated ML trees and seven coalescent species trees and visualized as pie charts showing the proportion of trees supporting each branch (Fig. 3a). In this approach, each inferred tree is treated as a “locus,” adapting the conceptual framework of gene concordance factors (gCFs) to derive tCFs. We observed uniformly high tCFs for several clades defined in our study, Vinciguerriidae, Yarrellidae, and Phosichthyidae each reached 100% concordance, while Gonostomatidae attained 92%. Our revised Stomiidae exhibited 77% concordant splits (23% in the primary alternative), and Diplophidae showed 62% concordance (38% alternative). Notably, Sternoptychidae, despite strong bootstrap support under concatenation, displayed only 38% concordance, reflecting its consistent paraphyly in coalescent‐based trees (Fig. S1). By integrating the signal across methodological and dataset variations, tCFs provide a robust, quantitative measure of both support and conflict in our novel eight-family classification.

### Topology testing against alternative classifications

To evaluate the robustness of our proposed eight-family classification against the recently proposed three-family framework by Smith et al. [13]. We conducted Approximately Unbiased (AU) topology tests in IQ-TREE using the 936-locus alignment for both the 64-taxon and 135-taxon datasets. Although our dataset does not statistically reject the monophyly of Stomiidae sensu Smith et al. [13] (*p* > 0.05), this topology was only ever resolved in one of the 13 species trees (six concatenation-based ML and seven coalescent-based) inferred in our study (Table 1; Fig. S1). Recognizing a monophyletic Stomiidae sensu Smith et al. [13] in our trees would collapse most Stomiiform genera into a single, overly broad Stomiidae. In contrast, our eight-family classification would be more informative and robust, even under topological uncertainty, with high tree concordance factors (tCFs) for most families (e.g., 100% for Vinciguerriidae, Yarrellidae, and Phosichthyidae; 92% for Gonostomatidae) (Fig. 3a). Furthermore, when our eight families are mapped onto the ultraconserved element (UCE) and morphological tree of Smith et al. [13], seven of our eight families remain monophyletic, providing additional evidence of their stability and robustness to topological variation.

### Proposed taxonomic classification

Our current classification framework integrates molecular and morphological data to establish a robust taxonomy. We compiled a morphological character matrix (Fig. S4) using traits from Harold & Weitzman (1996) [15], with further validation for some [13, 16, 17, 19]. These traits were mapped onto the ML phylogenetic tree derived from our genomic data (Fig. 3a). We then applied ACCTRAN and DELTRAN character optimizations methods to infer ancestral states on the tree. ACCTRAN prioritizes changes closer to the root, favoring early trait gains and subsequent reversals, whereas DELTRAN shifts changes toward the tips, emphasizing parallel trait evolution (Fig. S5).

**Family Diplophidae** Fowler 1925

**Type genus:***Diplophos* Günther 1873

**Diversity.** 9 species in 2 genera.

**Diagnosis.** Diplophidae is unique among stomiiform families in possessing photophore rows on the lower jaw and lacking pleural ribs associated with the third vertebra. It is further diagnosed by a high vertebral count (44–94 in diplophids vs. 28–60 in other stomiiforms) and a high ventral photophotore count (70–115 vs. 19–95); a well-developed median adductor mandibulae divided into two distinct muscles (vs. reduced in all other stomiiforms); a horizontally oriented extensor proprius pelvicus (vs. vertically-oriented in all other stomiiforms); a large nasal opening extending anteriorly to at least the nasal capsule (vs. small in other stomiiformes); a flattened, dentigerous, and horizontally-oriented basihyal (vs. cylindrical, edentate, and vertically-oriented in all other stomiiforms); and a ventrally bifurcated first pharyngobranchial shaft (vs. non-bifurcated in all other stomiiforms) (Additional file 4).

**Genera included.***Diplophos* (7 spp.) and *Manducus* (2 spp.).

**Comments.** The subfamily name “Diplophinae” was first proposed to accommodate the “greatly elongate” and “band-like” body plan of the genus *Diplophos* [26]. Shortly after, this genus was then reclassified as a maurolicid, and then a gonostomatid. Approximately thirty years later, “Manducinae” was similarly proposed to house *Manducus* and three other genera [27]. However, both subfamilial classifications failed to be considered and *Diplophos* and *Manducus* are currently recognized within Gonostomatidae. Subsequent phylogenetic accounts have varied in their treatments of *Diplophos* and *Manducus,* with some studies considering both genera as early-branching stomiiforms [17], or even sister to all other stomiiforms. The name “Diplophidae” was first acknowledged in 2004 [28], but remained controversial and was not widely recognized [3]. Our study corroborates previous analyses, with *Diplophos* sister to all other stomiiforms except *Vinciguerria,* and acknowledges the family Diplophidae as its own lineage, based on morphological and molecular evidence (more details in Supplementary Material).

**English name.** Portholefishes.

**Family Gonostomatidae** Cocco 1838

**Type genus:***Gonostoma* Rafinesque 1810

**Diversity.** 25 species in 6 genera.

**Diagnosis.** Gonostomatidae is diagnosed by a unique combination of traits. The family differs from all stomiiform families except Diplophidae in having beta-type photophores (vs. alpha or gamma in other stomiiforms) and in the presence of an accessory neural arch (vs. absent in other stomiiforms). Gonostomatidae differs from Diplophidae by the following characters: absence of photophore rows on the lower jaw (vs. presence in diplophids); presence of pleural ribs associated with the third vertebra (vs. absence in diplophids); reduction of one or both sections of the median adductor mandibulae (vs. well-developed in diplophids); a vertically-oriented extensor proprius pelvicus (vs. horizontally-oriented in diplophids); a small nasal opening not extending to the nasal capsule (vs. a large and anteriorly-extending nasal in diplophids); a cylindrical, edentate, and vertically-oriented basihyal (vs. flattened, dentigerous, and horizontally-oriented in diplophids); and a non-bifurcated first pharyngobranchial shaft (vs. ventrally-bifurcated in diplophids) (Additional file 4).

**Genera included.***Cyclothone* (14 spp.), *Gonostoma* (2 spp.), *Margrethia* (2 spp.), *Sigmops* (5 spp.), *Triplophos* (1 sp.), and *Zaphotias* (1 sp.).

**Comments.** Within the family, morphological and molecular analyses have reliably supported a monophyletic clade composed of *Cyclothone*, *Gonostoma*, *Margrethia*, *Sigmops* (sometimes as a synonym of *Gonostoma*) and *Zaphotias* (previously *Bonapartia*) [9, 13, 15]. We find Gonostomatidae to include these five genera and the additional genus *Triplophos*, which has been considered an early-branching gonostomatid [9] or an ally of *Diplophos* or the phosichthyids [15] owing to its elongate body and a series of unusual osteological characteristics. Molecular studies have been highly variable in their treatment of *Triplophos*, with various analyses placing it in Sternoptychidae [29], sister to all stomiiforms besides *Vinciguerria* and the other gonostomatids [8], or sister to the phosichthyid-stomiid clade “Photichthya” [13]. Our study is unique in resolving *Triplophos* within a monophyletic Gonostomatidae, and sister to *Zaphotias*. As such, we recognize six genera within Gonostomatidae.

**English name.** Bristlemouths. Includes also fangjaws (*Gonostoma*, *Sigmops*).

**Family Ichthyococcidae fam nov**.

**Type genus** (by monotypy)**:***Ichthyococcus* Bonaparte 1840

**Diversity.** 7 species in 1 genus.

**Diagnosis.** Ichthyococcidae is unique among stomiiform families in lacking an alveolar process, having the maxilla fused to the anterior supramaxilla, and showing reduction of the premaxilla and the apophyses of the first vertebra. It is further defined by a unique combination of characters. The family differs from all other families except Phosichthyidae (sensu present study) and Stomiidae in having the anal-fin origin posterior to dorsal fin (vs. below or anterior to dorsal fin in other stomiiforms); the bases of the posterior four branchiostegal rays crowded together (vs. widely separated); a reduced mesopterygoid (vs. well developed); and an ascending process of the premaxillary symphysis with straight medial surfaces (vs. curved medial surfaces). Ichthyococcidae further differs from Phosichthyidae and Stomiidae in having a well-developed posterior palatine process (vs. significantly reduced); variably-sized medial jaw teeth (vs. mainly large medial jaw teeth); more than six posterior ceratohyal branchiostegal rays; and fewer than 14 branchiostegal photophores (Additional file 4).

**Genera included.***Ichthyococcus* (7 spp.).

**Comments.** The phylogenetic placement of genus *Ichthyococcus* has been historically contentious, but has not changed significantly since its incorporation into Weitzman’s “Phosichthyidae” in the late twentieth century [30]. Since then, *Ichthyococcus* has been recurrently identified as an outlier within the family, due to its stout body and a number of specialized characteristics, including a beaked mouth and complex sensory structures absent in other phosichthyids [15, 30]. Morphological and molecular analyses have consistently identified strong support for a clade containing *Ichthyococcus*, *Phosichthys*, *Woodsia*, and Stomiidae [8, 15, 29]. Our phylogeny produces identical results, with *Ichthyococcus* sister to the remaining genera and family mentioned above. Based on previous phylogenomic analyses and distinct characteristics, we recognize a new monotypic family, Ichthyococcidae, to accommodate the genus *Ichthyococcus* (more details in the Supplementary Material).

**English name.** Fireflyfishes.

Note: Despite morphological divergence from the “true” lightfishes of genera *Phosichthys* and *Woodsia*, species of *Ichthyococcus* have historically also been called “lightfishes.” To distinguish them from the Phosichthyids, the authors propose the new English name “fireflyfishes,” in reference to their small, compact bodies and bright ventral photophores.

**Family Phosichthyidae** Weitzman 1974

**Type genus:***Phosichthys* Hutton 1872

**Diversity.** 3 species in 2 genera.

**Diagnosis.** Phosichthyidae is defined by a unique combination of traits. This family differs from all other stomiiform families except Stomiidae in having a reduced posterior palatine process (vs. well developed in other stomiiforms); mainly large medial jaw teeth (vs. variably sized); six or fewer posterior ceratohyal branchiostegal rays; and 14 or more branchiostegal photophores. Phosichthyidae further differs from Stomiidae in having the first vertebral parapophyses longer than the second (vs. shorter in stomiids); lacking an ossified Baudelot’s ligament (vs. both shrunken and with ossified Baudelot’s ligament in stomiids); and epineurals fused to neural arches for less than half of the body length (vs. more than half in stomiids) (Additional file 4).

**Genera included.***Phosichthys* (1 sp.) and *Woodsia* (2 spp.).

**Comments.** The family “Phosichthyidae” was initially used to categorize seven morphologically-divergent gonostomatid genera—*Ichthyococcus*, *Phosichthys*, *Pollichthys*, *Polymetme*, *Vinciguerria*, *Woodsia*, and *Yarrella* (Table 2)—characterized by an uneven mixture of anatomical traits typical of both stomiid and non-stomiid families [30]. Anatomical similarities between these genera are tenuous, and morphological analyses have resolved the “phosichthyid” genera as representing up to six different clades within Stomiiformes [15]. Molecular studies have strongly supported this potential non-monophyly, with the seven constituent genera being resolved as two [9], three [8, 13], or four [29] clades, depending on taxon sampling size. A sister relationship between the genera *Phosichthys* and *Woodsia* is well-supported by both molecular and morphological evidence [8, 13, 29, 30], including herein. Therefore, we redefine Phosichthyidae to contain only two genera, *Phosichthys* and *Woodsia*, with no subfamily-level taxa. The remaining five “phosichthyid” genera—*Ichthyococcus*, *Pollichthys*, *Polymetme*, *Vinciguerria*, and *Yarrella*—based on morphological and molecular analyses, do not form a clade with *Phosichthys* and *Woodsia*, and are therefore acknowledged within other families herein.

**English name.** Lightfishes.

**Family Sternoptychidae** Duméril 1805

**Type genus:***Sternoptyx* Hermann 1781

**Diversity.** 79 species in 10 genera.

**Diagnosis.** Sternoptychidae is unique among other stomiiform families in having a single epural; fusion of the third and fourth hypurals; alpha-type photophores; and shortened, subequal parapophyses of the first two vertebrae without an ossified Baudelot’s ligament. The family is further defined by a unique combination of characters. Sternoptychidae shares with some gonostomatids a lateral adductor mandibulae subdivided into dorsal and ventral sections (vs. undivided in other stomiiforms) and protracted photophore metamorphosis (vs. rapid). Sternoptychidae is distinguishable from these gonostomatids by the presence of an ossified accessory neural arch (Additional file 4).

**Genera included.***Araiophos* (2 spp.), *Argyripnus* (9 spp.), *Argyropelecus* (7 spp.), *Danaphos* (2 spp.), *Maurolicus* (15 spp.), *Polyipnus* (34 spp.), *Sonoda* (2 spp.), *Sternoptyx* (4 spp.), *Thorophos* (2 spp.), and *Valenciennellus* (2 spp.).

**Comments.** Sternoptychidae has been widely recognized as a distinct and monophyletic clade since 1974. This family was formed by the unification of three deep-bodied (“sternoptychine”) and seven shallow-bodied (“maurolicine”) stomiiform genera into a single taxon. Most molecular studies have supported the monophyly of this pairing, with the exception of some topological flukes (e.g., the exclusion of *Valenciennellus* or inclusion of *Triplophos*, neither of which have been replicated in subsequent analyses) [8, 9, 13, 31]. Traditionally, Sternoptychidae has been divided into two subfamilies, Sternoptychinae and Maurolicinae (a third, Polyipninae, was proposed in the nineteenth century [32], but was subsumed into Sternoptychinae). Evidence for this two-subfamily system has been intermittent, parsimony analyses of morphology suggested a paraphyly of Maurolicinae with respect to Sternoptychinae [15], and molecular analyses have often found the opposite (paraphyly of Sternoptychinae with respect to Maurolicinae) [9, 29, 31]. Our study supports the latter scenario, the sternoptychine genus *Polyipnus* forms a low-support clade with the maurolicine genera, rendering Sternoptychinae paraphyletic. Still, our study is lacking data from three maurolicine genera (*Araiophos*, *Sonoda*, and *Thorophos*), which means the monophyly of Maurolicinae cannot be adequately evaluated. Owing to topological uncertainty across recent studies, and an absence of genetic data for important genera, we recommend the temporary discontinuation of subfamily-level taxa in Sternoptychidae, until advanced techniques with further taxon sampling are able to confidently clarify genus-level relationships. We adopt the traditional ten-genus definition of Sternoptychidae, with no subfamily-level classifications.

**English name.** Hatchetfishes (*Argyropelecus*, *Polyipnus, Sternoptyx*); pearlsides (*Araiophos*, *Argyripnus*, *Maurolicus*, *Sonoda*, *Thorophos*); bottlelights (*Danaphos*); and constellationfishes (*Valenciennellus*).

**Family Stomiidae** Bleeker 1859

**Type genus:***Stomias* Jordan & Seale 1906

**Diversity.** 327 species in 27 genera.

**Diagnosis.** Stomiidae is unique among stomiiform families in having an ossified Baudelot’s ligament on the first two vertebral parapophyses; lacking gill rakers in adults; absence of the sixth hypural; division of the geniohyoideus into dorsal and ventral portions; and anterior and posterior enlargement of the posterior pelvic plate, with a cartilaginous core extending posteriorly beyond the ossified portion of the plate (Additional file 4).

**Genera included.***Aristostomias* (6 spp.), *Astronesthes* (50 spp.), *Bathophilus* (20 spp.), *Borostomias* (6 spp.), *Chauliodus* (9 spp.), *Chirostomias* (1 sp.), *Echiostoma* (1 sp.), *Eupogonesthes* (1 sp.), *Eustomias* (134 spp.), *Flagellostomias* (1 sp.), *Grammatostomias* (4 spp.), *Heterophotus* (1 sp.), *Idiacanthus* (3 spp.), *Leptostomias* (12 spp.), *Malacosteus* (2 spp.), *Melanostomias* (18 spp.), *Neonesthes* (2 spp.), *Odontostomias* (2 spp.), *Opostomias* (2 spp.), *Pachystomias* (1 sp.), *Photonectes* (29 spp.), *Photostomias* (6 spp.), *Rhadinesthes* (1 sp.), *Stomias* (12 spp.), *Tactostoma* (1 sp.), *Thysanactis* (1 sp.), and *Trigonolampa* (1 sp.).

**Comments.** The historical delimitations of family Stomiidae have been largely uncontroversial for forty years, since the demotion of six families (Astronesthidae, Chauliodontidae, Idiacanthidae, Malacosteidae, Melanostomiidae, and Stomiidae) into subfamilies within Stomiidae [16]. Early molecular investigations have occasionally failed to resolve a monophyletic Stomiidae due to the exclusion of the subfamily Chauliodontinae [8, 29]. However, phylogenomic investigations, present study included, reliably recover a monophyletic and well-supported Stomiidae that includes the chauliodontines [9, 13]. Unlike other studies [8, 16], our results discourage the use of these subfamily-level classifications. The astronesthines are split across three lineages, the malacosteines across three more, and the melanostomiines across seven. The three remaining subfamilies — Chauliodontinae, Idiacanthinae, and Stomiinae — resolve as monophyletic in our phylogeny by virtue of monotypy. Due to the topological uncertainty and lack of resolution of the six-subfamily system across multiple studies, including herein, we recommend the recognition of a monophyletic Stomiidae, containing its 27 traditional genera, but we also disregard the Stomiidae sub-family system until adequate taxonomic sampling and consistent placement of stomiids within the family have been resolved (more details in the Supplementary Material).

**English name.** Dragonfishes. Includes also stareaters (*Astronesthes*); loosejaws (*Aristostomias*, *Grammatostomias*, *Malacosteus*, *Photostomias*); boafishes (*Stomias*); snaggletooths (*Borostomias*, *Eupogonesthes*, *Heterophotus*, *Neonesthes*, *Rhadinesthes*); viperfishes (*Chauliodus*); and sawtails (*Idiacanthus*).

**Family Vinciguerriidae fam. nov**.

**Type genus:***Vinciguerria* Jordan & Evermann 1896

**Diversity.** 6 species in 2 genera.

**Diagnosis.** Vinciguerriidae is unique among other stomiiform families in having an elongate hyomandibular spine bound to the surface of the mesopterygoid by a ligament (vs. short and detached from mesopterygoid in all other stomiiforms); fusion or tight adherence of the second basibranchial tooth plates (vs. absent or loosely adhering in other stomiiforms); and close adherence of the third basibranchial tooth plates on the dorsal surface (vs. lateral to basibranchial). The family is further defined by a unique combination of traits. Vinciguerriidae differs from the other early-branching families (Diplophidae, Gonostomatidae, and Sternoptychidae) by presence of a posterior photophore; gamma-type photophores; an anteriorly angled dorsal uncinate process of the second pharyngobranchial (vs. straight and vertical in aforementioned families); and presence of a serial photophore duct and lumen.

Vinciguerriidae differs from Yarrellidae in having a posterior photophore; a-cell radiating configuration (vs. irregular in yarrellids); separate contralateral and ipsilateral branches of the premaxillary-rostrodermethmoid ligament (vs. fused in yarrellids); and presence of tooth plates on the fourth basibranchial. Lastly, Vinciguerriidae differs from the remaining families (Ichthyococcidae, Phosichthyidae, and Stomiidae) in having the anal fin originating below or anterior to the dorsal-fin origin (vs. posterior in aforementioned families); the posterior four branchiostegal separated from one another; an anterior palatomaxillary ligament looped over the dorsal surface of the lateral process of the rostrodermethmoid; a well-developed mesopterygoid; a medial concavity of the ascending process of the premaxillary symphysis; and the presence of tooth plates on the fourth basibranchial (Additional file 4).

**Genera included.***Pollichthys* (1 sp.) and *Vinciguerria* (5 spp.).

**Comments.***Vinciguerria* was initially described as a diminutive “maurolicid” in 1896 [33] but was eventually relocated into Gonostomatidae. In 1959, the morphologically similar *Pollichthys* was also described as a fellow gonostomatid [34], until both were eventually recognized within Phosichthyidae in 1974 [30]. Since then, their placement has remained contentious, though they reliably exhibit a sister relationship to one another [11, 15]. Further molecular analyses have frequently reaffirmed this relationship, in addition to the *Pollichthys* + *Vinciguerria* clade as early-branching within stomiiforms [8, 9, 13, 29]. Although our dataset only incorporated *Vinciguerria*, based on previous morphological and molecular studies, we are confident in hypothesizing a sister relationship between *Vinciguerria* and *Pollichthys* [13, 15]. We relocate both genera to the new family Vinciguerriidae, a morphologically distinct lineage of early-branching stomiiforms that has been reliably resolved across multiple studies.

**English name.** Lighthousefishes (*Vinciguerria*) and stareyes (*Pollichthys*).

**Family Yarrellidae fam. nov.**

**Type genus:***Yarrella* Goode & Bean 1896

**Diversity.** 8 species in 2 genera.

**Diagnosis.** Yarrellidae is unique among other stomiiform families in having the contralateral and ipsilateral branches of the premaxillary-rostrodermethmoid ligament fused into a continuous sheet of connective tissue (vs. unfused in all other stomiiforms). Yarrellidae is further defined by a unique combination of characters. The family differs from Diplophidae, Gonostomatidae, and Sternoptychidae by the presence of a second epural (vs. absence in those families) and by having a gamma-type photophores (vs. alpha or beta). Yarrellidae is distinguishable from the remaining families (Ichthyococcidae, Phosichthyidae, Stomiidae, and Vinciguerriidae) by the absence of a posterior orbital/postorbital photophore (vs. presence in those families) and by the irregular configuration of photophore A cells (vs. regular) (Additional file 4).

**Genera included.***Polymetme* (6 spp.) and *Yarrella* (2 spp.).

**Comments.** The genera *Yarrella* and *Polymetme* were described in 1896 and 1926, respectively, as gonostomatids. They were relocated into Phosichthyidae in 1974 [30], where they were considered deeply divergent within the family. Morphological analyses subsequently found *Polymetme* forming a clade with all other phosichthyids and the family Stomiidae, to the exclusion of *Yarrella*. Past molecular analyses have also resolved a *Polymetme* + *Yarrella* sister relationship, which was in turn sister to a clade containing *Ichthyococcus*, *Phosichthys*, *Woodsia*, and the stomiids [8, 13, 15]. The repeated recovery of this clade leads us to support the recognition of a new family, Yarrellidae, for these two divergent genera, previously considered as phosichthyids (more details in Supplementary Material).

**English name.** Rendezvousfishes.

## Discussion

Our phylogenomic analysis of Stomiiformes provides a comprehensive framework for understanding the evolutionary relationships within this diverse order, resolving long-standing questions while also highlighting areas of the phylogeny that remain unresolved and in need of taxonomic revision. The consistent topologies and higher-level clades derived from concatenation and supermatrix methods (Fig. 2; Fig. 3) demonstrate the efficacy of these approaches in handling high levels of missing data [35, 43], a finding consistent with previous studies that highlight the minimal impact of incomplete taxa or markers on phylogenetic accuracy [36–38]. Especially within sufficiently large datasets, this robustness is attributed to the low sampling error associated with matrices containing a large number of genome-wide markers, which provide sufficient phylogenetic signal to mitigate potential biases [38–40]. In addition, our extensive taxonomic sampling includes representatives from all recognized families and approximately 70% of the genera within Stomiiformes. Our results support the early-branching position of *Vinciguerria*, consistent with recent studies [8, 25], while challenging the traditional placement of Gonostomatidae as sister to all other stomiiforms [9, 12, 30]. This reveals a more complex evolutionary history and prompted a revised classification of the order that better reflects these phylogenetic relationships.

A critical finding of our study is the polyphyly of the traditional Phosichthyidae, with its seven genera dispersed across four independent lineages. Three lineages resolved as sister groups to a monophyletic Stomiidae (Table 1; Fig. 2), while the fourth lineage is resolved as the earliest-branching stomiiform lineage. This topology, consistently supported across our analyses (Fig. 3a, b, c) directly contradicts the broadly circumscribed, monophyletic Stomiidae proposed by Smith et al. [13], which encompasses 35 genera (344 species), including all phosichthyids and the gonostomatid genus *Triplophos*. Importantly, their definition of Stomiidae is not resolved as monophyletic in our trees due to the position of *Vinciguerria* as the sister to all other Stomiiformes (Table 1; Fig. 3a, b). Moreover, the genera Smith et al. [13] incorporates into Stomiidae, such as *Triplophos*, *Ichthyococcus*, and *Phosichthys*, represent distinct lineages from the traditionally-defined stomiids and possess well-defined morphological synapomorphies, such as the specialized serial photophore ducts in *Phosichthys* and the extreme upper jaw modifications in *Ichthyococcus* (Additional file 4). These findings inform our proposed recognition of eight monophyletic families, which better reflects the phylogenetic structure and morphological diversity within Stomiiformes.

The proposed classification of Stomiiformes into eight families, Vinciguerriidae, Diplophidae, Gonostomatidae, Sternoptychidae, Yarrellidae, Ichthyococcidae, Phosichthyidae, and Stomiidae, captures the deep evolutionary divergences and ecological adaptations within the order (Additional file 4). Each newly recognized family exhibits clear diagnostic features that reflect distinct evolutionary trajectories. For instance, Vinciguerriidae is characterized by an elongated hyomandibular spine bound to the mesopterygoid and a unique configuration of pharyngobranchial tooth plates, while Diplophidae exhibits an exceptionally high vertebral count (44–94 versus 28–60 in other stomiiforms), a divided median adductor mandibulae, and a distinctive row of photophores on the ventral side of the lower jaw [15, 17, 19]. Notably, the resurrection of Diplophidae is warranted by the consistent placement of *Diplophos* as sister to the remaining gonostomatids and other stomiiforms (excluding the genus *Vinciguerria*), challenging its traditional inclusion within Gonostomatidae. Similarly, Yarrellidae displays unique ligament architecture, including the fusion of the premaxillary-rostrodermethmoid ligaments, alongside their distinctive gamma-type photophores and rapid metamorphosis of light organs [15].

Our classification of Stomiiformes into eight monophyletic families adheres to the principles of phylogenetic taxonomy and the International Code of Zoological Nomenclature by recognizing monophyletic groups defined by clear diagnostic traits, offering a framework that reflects the anatomical innovations that have potentially favored stomiiform diversification across mesopelagic niches. For instance, traits such as specialized sensory structures in Ichthyococcidae, unique photophore anatomy in Sternoptychidae, and distinct skeletal architecture in Vinciguerriidae are just a few unique adaptations that highlight an evolutionary distinctiveness that was previously obscured behind large and unstable taxon names defined by misleading superficial similarities (Additional file 4). There is risk of oversimplifying complex relationships by collapsing diverse lineages into a single, non-monophyletic Stomiidae [13], reducing the phylogenetic informativeness of the taxon and obscuring unique evolutionary histories. Our recognition of eight families addresses this concern by reflecting natural diversification patterns and enhancing the predictive power for ecological and morphological studies. While we align with Smith et al. [13] in rejecting the traditional four family system of Stomiiformes (Fig. 2; Fig. 3a, b), our classification provides a more robust phylogenetic framework, supported by consistent phylogenetic resolution (Fig. 3a, b) and detailed morphological data (Additional file 4). This revised classification offers a robust foundation for future research into the ecological and evolutionary dynamics of deep-sea fishes, facilitating precise investigations into their vertical habitat use, feeding strategies, and evolutionary dynamics within the mesopelagic and bathypelagic zone [2, 40, 41].

## Conclusion

Our comprehensive phylogenomic analysis of the order Stomiiformes significantly refines the resolution of inter- and intrafamilial relationships within this group. Although the taxonomic arrangement of stomiiform families in our phylogenies diverges from both traditional and contemporary systematic frameworks, the molecular phylogenetic evidence, bolstered by morphological characters, provides robust support for our revised classifications. Additionally, we advocate the adoption of rigorous QC protocols, when integrating sequences from BOLD or GenBank to ensure data reliability. Nevertheless, fully elucidating the internal relationships, particularly within Stomiidae, will necessitate further investigation with broader taxonomic sampling and an expanded set of genome-wide markers. This study underscores the critical role of extensive taxonomic representation and stringent QC measures in resolving contentious evolutionary relationships, while establishing a foundation for future explorations into the evolutionary history of Stomiiformes.

## Methods

### Taxonomic sampling

Our taxonomic sampling follows a two-step approach. First, we subsampled tissue samples from museum collections for 44 stomiiform species and augmented this dataset by incorporating 27 additional stomiiform species with genome skimming data available from NCBI. Following QC analyses, as outlined in Arcila et al. [42], and after removing duplicate species, our Stomiiformes genomic dataset comprised 60 species, including 31 of the 52 recognized genera and all historically and currently recognized families. Second, we retrieved 2,474 COI sequences from BOLD. We estimated a preliminary tree in FastTree and identified clusters of sequences with the same name, which we refer to as ‘bins’. We identified 181 stomiiform bins, however, some sequences could not be accurately assigned to any bin and are listed as ‘ID cannot be determined’ (Fig. S2; Additional file 3). After COI quality control and integration with the genome-wide dataset, our taxonomic sampling consisted of 135 of 464 valid stomiiform species (Additional file 2). For outgroup selection, molecular phylogenetic analyses have consistently resolved Osmeriformes (smelts, icefishes, marine minnows, and sweetfishes) as the sister group of Stomiiformes, forming the subcohort Stomiatti [9, 10, 12, 41, 42, 44]. Based on these well-supported relationships, we selected four osmeriform species as outgroups: *Retropinna tasmanica* (Retropinnidae)*, Osmerus mordax* (Osmeridae)*, Salanx* sp. (Salangidae)*,* and *Plecoglossus altivelis* (Plecoglossidae), representing all currently recognized families within the osmeriform order.

### Exon-capture sequencing, data assembly, and alignment

For our newly generated data, we extracted genomic DNA from fin or muscle tissue using a phenol–chloroform protocol in a 96-well plate format on a GenePrep (Autogen Inc.) platform following manufacturer’s instructions at the Laboratories of Analytical Biology at the Smithsonian Institution’s National Museum of Natural History. We performed a QC assessment of the extractions by running 1μL of eluted DNA on a 1.0 agarose gel stained with GelRed (Biotium) and by visually inspecting whether bands of high molecular weight DNA were visible. Using exon capture approaches, we sequenced 1104 single-copy markers with the “Backbone 1” probe set of Hughes et al. [44], including 17 additional legacy nuclear (e.g., ENC1, PLAGL2, and MYH6), and 10 mitochondrial (mtDNA) markers commonly used in fish systematics. For data assembly, low-quality bases and contaminated adapter sequences were trimmed using Trimmomatic v0.36 54 [10]. These reads were then mapped to their reference sequences for all exons in our probe set spanning all ray-finned fishes using the Burrows-Wheeler Alignment (BWA-MEM) tool, and SAMtools v.1.7–1.9 [10]. We then assembled the mapped reads for each exon using Velvet v1.2.10.

We assembled Velvet-generated contigs using the automated target-restricted method (aTRAM) software with Trinity v2.8.5 [10] as the assembler. We were able to remove the number of redundant contigs as well as any contigs lacking open reading frames using CD-HIT-EST [10] and Exonerate, respectively. The filtered gene pre-alignments were combined with orthologous exon markers mined from publicly available raw sequence reads after undergoing the same assembling protocol. Next, the combined exon sequences were aligned using the Multiple Alignment of Coding Sequences (MACSE v2.05) program. Frameshifts and stop codons were accounted for by MACSE, and the alignments were filtered, removing single-taxon insertions, highly gapped edges, and short sequences using a cleaning python script (AlignmentCleanerCodons.py) provided in the Hughes et al. [10] pipeline. Our final genetic dataset consisted of 936 nuclear and one mtDNA marker. The alignments were manually inspected and adjusted as needed to preserve correct reading frames. Following inspection, we removed low-quality reads and flanking regions while correcting potential misalignments using Geneious Prime v2024.0.7. Lastly, all gene alignments were concatenated into a super-matrix using the python package, AMAS [10].

### Incorporating publicly available sequence data and quality control

For the genome skimming data available in NCBI, we extracted 977 of the 1,104 exon markers using the Hughes et al. (2018) bioinformatic pipeline [10]. These sequences were filtered down to 936 exons, then combined with our exon-capture data. Sequence alignments were performed using MACSE v2.05 and concatenated using AMAS [10]. To assess the quality of the NCBI-derived species*,* we conducted ML analyses using FastTree [45], followed by a visual inspection. Six stomiiform individuals were removed from our combined genomic data set due to extensive missing data or potential identification issues. To incorporate mtDNA sequences into our final dataset, we initially compiled all available COI sequences from BOLD, focusing exclusively on Stomiiformes, alongside COI data from 40 of the 60 species in our genomic dataset (49 of which included COI sequences). These COI sequences were aligned using MACSE v2.05 [10]. Given evidence of high amounts of potential misidentifications in public repository data [46, 47], we implemented QC through manual inspection in Geneious and ML analyses with FastTree. Based on phylogenetic placement and clustering patterns supported by two or more individuals, we incorporated 86 additional COI-only species into our genomic dataset. The resulting expanded dataset comprises one representative per species, totaling 135 taxa, including 131 Stomiiformes and four outgroup taxa.

### Maximum likelihood phylogenetic analysis

We conducted concatenation-based phylogenomic analyses on both the genomic (936 nuclear markers, 64 species) and expanded (936 nuclear markers + COI, 135 species) datasets. Gene alignments were concatenated using AMAS. Initial ML trees were estimated using FastTree to identify and remove contaminations, misidentified taxa, and duplicate specimens. Final ML trees were estimated using IQ-TREE v2.026 [48] under the mixture model "MIX {JC, K2P, HKY, GTR}", which optimizes substitution models for individual sites. Node support was assessed using 1,000 ultrafast bootstrap approximation (UFBoot2) [42], which provides robust branch support values with reduced computational demand [42, 49].

To evaluate topological consistency in our nuclear dataset, we partitioned the 936 loci into three non-overlapping subsets using a custom R script with similar gene length and parsimony-informative sites while ensuring representation of all 64 taxa. From the 936 nuclear loci, we excluded nine anchor exons representing all 64 taxa, yielding 927 exons. These were divided into three subsets of 309 exons each. The nine anchor exons were then reintroduced to each subset, resulting in three datasets of 318 exons each (Additional file 1). No COI data were included in the nuclear subset analyses. Data properties and filtering criteria are detailed in Additional file1: Table S1. To further test for the phylogenetic robustness of our genomic dataset, we constructed three additional datasets which attempted to account for missing data within our matrices. A dataset including only genes with a sequence length greater than 250 bps, a dataset including only genes representing more than 20 taxa, and a dataset including only genes representing more than 35 taxa. Each subset and dataset was analyzed using IQ-TREE and followed the concatenation protocol described above.

### Coalescent-based species tree analysis

We performed coalescent-based phylogenomic analyses using our 936 nuclear loci and COI data for all 64 species. Gene alignments with fewer than four sequences were excluded (n = 37), resulting in 899 nuclear loci and COI for coalescent analyses. Individual gene trees were estimated using IQ-TREE under the mixture model with 1,000 UFBoot2 replicates as above. To minimize gene tree estimation error, which can bias species tree inference [50–52], we generated two additional gene tree sets by collapsing branches with support values below 10% and 30% using Newick Utilities. These thresholds were selected based on preliminary analyses testing collapse values from 0 to 50%. Species trees were inferred using ASTRAL-IV [50], a summary coalescent species tree method to account for incomplete lineage sorting [51, 53]. To further assess clade resolution and topological consistency, we partitioned the 899 nuclear loci into three non-overlapping subsets using the above custom R script. Each subset included nine “anchor” loci, selected for complete taxon coverage and phylogenetic informativeness as described previously, while the remaining 890 non-anchor loci were evenly distributed across subsets. COI was then added into each subset, forming two subsets of 307 genes and one of 306 genes. Each subset was analyzed using ASTRAL-IV.

### Phylogenetic concordance and topology testing

To address the limitations of bootstrap support (BS) in large phylogenomic datasets [42] and to mitigate high gene-tree error estimation, we extended the concordance-factor framework to infer tree concordance factors (tCFs) using PhyParts [54]. In this approach, each species tree (rather than each gene tree) is treated as an independent “locus.” We assembled thirteen input topologies (six concatenated ML trees inferred with IQ-TREE v2.2.0 and seven multispecies-coalescent trees estimated with ASTRAL-IV) and designated our best-supported concatenated ML inference as the reference. All trees were rooted to the chosen outgroup prior to analysis. PhyParts computes, for each node in the reference topology, the proportion of input trees recovering the same bipartition (concordant), the proportion supporting the most frequent alternative split, and the remainder as other conflicts. These concordance and conflict metrics were rendered as proportional pie charts using the PhyPartsPieCharts python script. To further explore topological robustness, we compared our reference concatenated phylogeny against the recently proposed Stomiiformes classification [13] via Approximately Unbiased (AU) tests in IQ-TREE v2.2.0 on the 936-marker alignment under both the 135-taxon and 64-taxon samplings.

## Supplementary Information

Additional file 1. Summary of dataset properties, including the three phylogenomic approaches, all datasets used in this study, and their construction. We also provide the contents of each dataset, including gene alignments, the number of estimated gene trees, sequence alignment lengths, number of taxa, and the proportion of missing data.

Additional file 2. Maximum likelihood tree using FastTree containing 2525 individuals and used for sequence quality assessment. 2474 represented solely by COI data and 51 genome-wide sequences (colored red). Clades were placed into ‘bins’ with bin names representative of a species name. 181 bins were procured, and COI sequences were colored based on families. COI data was acquired from BOLD (https://v3.boldsystems.org/).

Additional file 3. Sequence quality assessment table for all COI individuals in this study.

Additional file 4. A summary table depicting all previously diagnosed morphological characters of Stomiiformes. The diagnosed numbers next to the related character trait is consistent with the diagnostic traits seen in other figures.

Additional file 5. 

## Data Availability

All data generated or analyzed during this study are included in this published article and in its supplementary information files. All concatenated sequence alignments, tree files, and custom R scripts used in this study are also available in a Dryad repository at http://datadryad.org/share/KpAnLfRYocgSJmTIgR_q0IXEiQzos4wVUt26wCB6Tlo.

## Abbreviations

AMAS: Alignment Manipulation and Summary
ASTRAL: Accurate Species Tree Algorithm
aTRAM: Automated Target Restricted Assembly Method
BOLD: Barcode of Life Data Systems
CD-HIT-EST: Cluster Database at High Identity with Tolerance – Expressed Sequence Tags
COI: Cytochrome C oxidase subunit 1
MACSE: Multiple Alignment of Coding Sequences
ML: Maximum likelihood
mtDNA: Mitochondrial deoxyribonucleic acid
NCBI: National Center of Biotechnology Information
QC: Quality control
