# Supplementary material for: Genome-wide phylogeny reshapes our understanding of the evolution of deep-sea dragonfishes, bristlemouths, viperfishes, and allies (Stomiiformes)
Source: BMC Ecol Evol. 2025 Oct 21;25:111. doi: 10.1186/s12862-025-02453-0 (PMC12542184; doi:10.1186/s12862-025-02453-0)
Supplement: Supplementary file 5 — Additional file 5. [file 12862_2025_2453_MOESM5_ESM.docx]

**Supplementary Material:**

**Genome-scale phylogeny reshapes our understanding of the evolution of deep-sea dragonfishes, bristlemouths, viperfishes, and allies (Stomiiformes)**

Solomon Chang^1^, Zach Heiple^1^, Delson Hays^1^, Fernando Melendez^1^, Casey Lee^1^, Ricardo Betancur-R.^1^, Dahiana Arcila^1*^

^1^Scripps Institution of Oceanography, University of California San Diego, 9500 Gilman Drive, La Jolla, CA 92093-0244, United States of America

*Corresponding author. Email: dkarcila@ucsd.edu

**Expanded Stomiiformes Classification Diagnosis:**

**Family Diplophidae** Fowler 1925

**Type genus**: *Diplophos* Günther 1873

**Diversity.** 9 species in 2 genera.

**Diagnosis.** Diplophidae is unique among stomiiform families in the presence of photophore rows on the lower jaw, and the absence of pleural ribs associated with the third vertebra. It is further diagnosed by high vertebral count (44-94 in diplophids vs 28-60 in other stomiiforms) and high ventral photophotore count (70-115 vs 19-95); well-developed median adductor mandibulae divided into two distinct muscles (vs reduced in all other stomiiforms); horizontally-oriented extensor proprius pelvicus (vs vertically-oriented in all other stomiiforms); a large nasal extending anteriorly to at least the nasal capsule (vs small in other Stomiiformes); a flattened, dentigerous, and horizontally-oriented basihyal (vs cylindrical, edentate, and vertically-oriented in all other stomiiforms); and a ventrally-bifurcated first pharyngobranchial shaft (vs non-bifurcated in all other stomiiforms) (Additional file 4: Table S2).

**Genera included.** *Diplophos* (7 spp.) and *Manducus* (2 spp.).

**Comments.** The subfamily name “Diplophinae” was first proposed to accommodate the “greatly elongate” and “band-like” morphology of the genus *Diplophos* (26). Shortly after, this genus was then listed as a maurolicid, and then subsequently classified as a gonostomatid. A second subfamily, “Manducinae,” was proposed in 1958 (27), for the type genus *Manducus* as well as the genus *Valenciennellus*, and the genera *Polymetme* and *Yarrella*. However, both subfamilial classifications failed to be considered and *Diplophos* and *Manducus* were eventually relocated to Gonostomatidae (28).

Subsequent taxonomic and phylogenetic accounts of gonostomatid fishes have varied in their treatment of *Diplophos* and *Manducus*. The two genera — sometimes as only *Diplophos*, with *Manducus* a junior synonym — have historically been considered early-branching stomiiforms (17), or even the sister to all other members of the order (15), but a distinct family-group name was not applied until the twenty-first century. The name “Diplophidae,” elevated from subfamily to family, was first deployed in 2004 (29) but not officially described, and since then the taxon name has made recurrent appearances while remaining unaccepted in most major ichthyological resources (3).

When *Diplophos* has appeared in recent molecular phylogenies, it has been placed in one of two positions: sister to all other gonostomatids (13,21), or occupying a unique branch entirely (30). The present study supports the latter, with *Diplophos* sister to all other stomiiforms except *Vinciguerria*. This outcome, following several decades of uncertain and unstable placement, supports the recognition of family Diplophidae as a unique stomiiform lineage, morphologically distinct from the gonostomatids. Despite the paucity of molecular data for *Manducus*, we propose the provisional inclusion of this genus as well as *Diplophos*, owing to a degree of morphological similarity between the two so high that they have previously been considered a single genus. No subfamilies are recognized.

**English name.** Portholefishes.

**Family Gonostomatidae** Cocco 1838

**Type genus:** *Gonostoma* Rafinesque 1810

**Diversity.** 25 species in 6 genera.

**Diagnosis.** Gonostomatidae is defined by a unique combination of traits. Gonostomatidae differs from all stomiiform families except Diplophidae in possessing beta-type photophores (vs alpha or gamma in other stomiiforms); and presence of an accessory neural arch (vs absence in other stomiiforms). Gonostomatidae differs from Diplophidae in possessing: absence of photophore rows on the lower jaw (vs presence in diplophids); presence of pleural ribs associated with the third vertebra (vs absence in diplophids); reduction of one or both sections of the median adductor mandibulae (vs well-developed in diplophids); vertically-oriented extensor proprius pelvicus (vs horizontally-oriented in diplophids); a small nasal not extending to the nasal capsule (vs a large and anteriorly-extending nasal in diplophids); a cylindrical, edentate, and vertically-oriented basihyal (vs flattened, dentigerous, and horizontally-oriented in diplophids); and a non-bifurcated first pharyngobranchial shaft (vs ventrally-bifurcated in diplophids) (Additional file 4: Table S2).

**Genera included.** *Cyclothone* (14 spp.), *Gonostoma* (2 spp.), *Margrethia* (2 spp.), *Sigmops* (5 spp.), *Triplophos* (1 sp.), and *Zaphotias* (1 sp.).

**Comments.** Gonostomatidae has occasionally served as a wastebasket taxon for stomiiform genera and only recently has its cladistic structure been elucidated in any detail. Nineteenth- and early twentieth-century definitions of the family included many genera later treated as phosichthyids or sternoptychids (28), and only in the last fifty years has Gonostomatidae been defined as a consistent set of eight genera. Two of those are herein relegated to the separate family Diplophidae, leaving only six genera in a revised Gonostomatidae following our proposed classification.

Since the 1970s, various analyses have corroborated the existence of a clade containing five traditional gonostomatid genera — *Cyclothone*, *Gonostoma*, *Margrethia*, *Sigmops* (sometimes considered a junior synonym of *Gonostoma*), and *Zaphotias* (=*Bonapartia*). Morphological analyses (15) have indicated strong support for the monophyly of this clade, as have molecular analyses (9,21). The present study reaffirms the monophyly of a gonostomatid clade containing these five genera. However, the traditionally-gonostomatid genera *Diplophos* and *Manducus* have been famously difficult to resolve and are not considered gonostomatids in the present study.

An eighth and final gonostomatid genus, *Triplophos*, has often been problematic within both molecular and morphological analyses of this family. The abnormal elongated body-plan, as compared to other gonostomatids, that is supposedly convergent with *Diplophos* and *Manducus*, has been previously labelled as either an early-branching gonostomatid (28), a phosichthyids, or an ally of *Diplophos* (15). Molecular analyses have included *Triplophos* among the sternoptychids (30); either resolving it sister to all other stomiiforms, except *Vinciguerria* and the other gonostomatids (8); or sister to the phosichthyid + stomiid clade (13). Our study is unique in resolving *Triplophos* within a monophyletic Gonostomatidae, as sister to *Zaphotias* (=*Bonapartia*). For now, the inclusion of *Triplophos* within Gonostomatidae is the best current option for this highly contentious genus. We would not be surprised if subsequent phylogenomic analyses resolve the genus *Triplophos* within a different clade of the stomiiform phylogeny. As such, we provisionally recommend the recognition of a monophyletic Gonostomatidae, reduced in size from eight genera to six, without subfamily-level taxa.

**English name.** Bristlemouths. Includes also fangjaws (*Gonostoma*, *Sigmops*).

**Family Ichthyococcidae fam nov.**

**Type genus** (by monotypy)**:** *Ichthyococcus* Bonaparte 1840

**Diversity.** 7 species in 1 genus.

**Diagnosis.** Ichthyococcidae is unique among stomiiform families in the absence of the alveolar process, the fusion of the maxilla to the anterior supramaxilla, and the reductions of the premaxilla and apophyses of the first vertebra.

This family is further defined by a unique combination of characters. Ichthyococcidae differs from all other families except Phosichthyidae (*sensu* present study) and Stomiidae: an anal fin origin posterior to dorsal fin (vs below or anterior to dorsal fin in other stomiiforms); bases of the posterior four branchiostegal rays crowded together (vs widely separated); a reduced mesopterygoid (vs well-developed); and ascending process of premaxillary symphysis with straight medial surfaces (vs curved medial surfaces). Ichthyococcidae differs from Phosichthyidae and Stomiidae in possessing: a well-developed posterior palatine process (vs significantly reduced in Phosichthyidae and Stomiidae); variably-sized medial jaw teeth (vs mainly large medial jaw teeth); more than six posterior ceratohyal branchiostegal rays; and fewer than fourteen branchiostegal photophores (Additional file 4: Table S2).

**Genera included.** *Ichthyococcus* (7 spp.).

**Comments.** After being dubiously placed in Sternoptychidae and then Gonostomatidae, the species-depauperate genus *Ichthyococcus* was ultimately incorporated into the new “Phosichthyidae” described in 1974 (28). Since the establishment of this family, the stout-bodied *Ichthyococcus* has been recurrently identified as an outlier due to a number of highly modified characteristics, including a complex beaked mouth and sensory structures not found in other genera of the same family (15,28).

Morphological analyses of *Ichthyococcus* have, for almost thirty years, identified a potential clade containing *Ichthyococcus*, *Phosichthys*, *Woodsia*, and Stomiidae (15), and early molecular studies have corroborated this finding (8,30). The present study concurs in resolving *Ichthyococcus* as a unique branch, sister to a clade containing the *Phosichthys-Woodsia* clade (Phosichthyidae *sensu* present study) and Stomiidae. This is also congruent with the recent morphological and combined molecular-morphological analyses (13). Based on ample previous evidence, *Ichthyococcus* is a unique and morphologically modified stomiiform lineage that we recognize as a new monotypic family, Ichthyococcidae.

**English name.** Fireflyfishes.

Note: Despite morphological divergence from the “true” lightfishes of genera *Phosichthys* and *Woodsia*, species of *Ichthyococcus* have historically also been called “lightfishes.” To distinguish them from the Phosichthyids, we propose the new English name “fireflyfishes,” in reference to their small, compact bodies and bright ventral photophores.

**Family Phosichthyidae** Weitzman 1974

**Type genus**: *Phosichthys* Hutton 1872

**Diversity.** 3 species in 2 genera.

**Diagnosis.** Phosichthyidae is defined by a unique combination of characters. Phosichthyidae differs from all families except Stomiidae in possessing: a reduced posterior palatine process (vs well-developed in other stomiiforms); mainly large medial jaw teeth (vs variably-sized); six or fewer posterior ceratohyal branchiostegal rays; and fourteen or more branchiostegal photophores. Phosichthyidae differs from Stomiidae by: the first vertebral parapophyses longer than the second, lacking an ossified Baudelot’s ligament (vs both shrunken and with ossified Baudelot’s ligament in stomiids); and epineurals fused to neural arches for less than half of the body length (vs more than half in stomiids) (Additional file 4: Table S2).

**Genera included.** *Phosichthys* (1 sp.) and *Woodsia* (2 spp.).

**Comments.** The family “Phosichthyidae” was described as a placeholder for seven morphologically-divergent gonostomatid genera — *Ichthyococcus*, *Phosichthys*, *Pollichthys*, *Polymetme*, *Vinciguerria*, *Woodsia*, and *Yarrella* — characterized by a mixture of traits typical of stomiids and those found in gonostomatids and sternoptychids (28). Subsequently, the monophyly of this family was refuted, and following studies have often opted to refer to the family as “‘Phosichthyidae’” or “the ‘phosichthyid’ genera” (15,17). A comprehensive osteological study of the family indicated a paraphyly with respect to Stomiidae, and that the “phosichthyid” genera may comprise as many as six distinct clades (15).

Suspicions of “phosichthyid” non-monophyly were abundantly reaffirmed in the early twenty-first century. The seven genera traditionally ascribed to this family have been resolved as two (9), three (8,13), or four separate clades (30) (as herein), heavily dependent on taxon sampling size. However, a sister relationship between the lightfish genera *Phosichthys* and *Woodsia* is observed whenever both genera are included (13), and morphological evidence indicates a high degree of similarity between the two. The remaining five “phosichthyid” genera — *Ichthyococcus*, *Pollichthys*, *Polymetme*, *Vinciguerria*, and *Yarrella* — do not form a clade with *Phosichthys* and *Woodsia* in any morphological or molecular analysis, present study included, and these genera are herein referred to other families.

Phylogenomic analyses have produced a high degree of topological convergence regarding the relationships between the “phosichthyid” and stomiid genera (13). The most recent work of Smith et al. (13) notably differs from all other taxonomic classifications in merging all former phosichthyid genera (as well as the gonostomatid *Triplophos*) into Stomiidae, producing a single ‘super’ family of 35 genera and 352 species. We disagree with this approach due to the lack of acknowledgement on their morphological and molecular diversity. We instead choose to split rather than merge, where we recognize four families, Ichthyococcidae, Phosichthyidae, Vinciguerriidae, and Yarrellidae,built from the former phosichthyid genera, in addition to a traditional 27-genus Stomiidae. With this current nomenclature, we are 1) generating a more accurate representation of their morphological and molecular distinction at the family-level, and 2) producing a taxonomic classification that will more easily accommodate potential future changes in the resolution of stomiiform clades.

**English name.** Lightfishes.

**Family Sternoptychidae** Duméril 1805

**Type genus**: *Sternoptyx* Hermann 1781

**Diversity.** 79 species in 10 genera.

**Diagnosis.** Sternoptychidae is unique among stomiiform families in the presence of a single epural, fusion of the third and fourth hypurals, alpha-type photophores, and shortened, subequal parapophyses of the first two vertebrae with no ossified Baudelot’s ligament. It is further defined by a unique combination of characters. Sternoptychidae shares with some gonostomatids: a lateral adductor mandibulae subdivided into dorsal and ventral sections (vs undivided in other stomiiforms) and protracted photophore metamorphosis (vs rapid). Sternoptychidae is distinguishable from these gonostomatids by the presence of an ossified accessory neural arch (Additional file 4: Table S2).

**Genera included.** *Araiophos* (2 spp.), *Argyripnus* (9 spp.), *Argyropelecus* (7 spp.), *Danaphos* (2 spp.), *Maurolicus* (15 spp.), *Polyipnus* (34 spp.), *Sonoda* (2 spp.), *Sternoptyx* (4 spp.), *Thorophos* (2 spp.), and *Valenciennellus* (2 spp.).

**Comments.** Sternoptychidae has been recognized in its current state since the late twentieth-century (28). The family was formed through unifying three deep-bodied (“sternoptychine”) and seven shallow-bodied (“maurolicine”) genera. The shallow-bodied genera had previously been assigned to their own family, Maurolicidae, after being initially described as gonostomatids. For the most part, subsequent studies have supported the monophyly of Sternoptychidae (8,9,13,31). The sternoptychid genus, *Valenciennellus*, has been resolved with the “phosichthyids” *Vinciguerria* and *Pollichthys* (30), while the “gonostomatid” *Triplophos* resolved within the sternoptychines in the same paper. Neither outcome has been replicated in subsequent phylogenies, and as such, we are inclined to recognize a monophyletic Sternoptychidae containing the 10 traditional genera.

Current taxonomy makes use of two sternoptychid subfamilies: Sternoptychinae and Maurolicinae (a third, Polyipninae, was proposed in 1893 (32) but failed to receive widespread endorsement despite topological uncertainty with regard to the deep-bodied type genus *Polyipnus*). Evidence for this two-subfamily system has been intermittent, a maximum-parsimony analysis based on morphological data presents Maurolicinae as paraphyletic with respect to the three sternoptychine genera, with *Thorophos* the first-branching sternoptychid genus (15). Later molecular analyses of only a few genera have found the opposite, *Maurolicus* nested within sternoptychine genera (sister to *Argyropelecus* (30), *Sternoptyx* (9), or a clade comprised of *Argyropelecus* and *Sternoptyx* (31)).

Our study resolves a paraphyletic Sternoptychinae with respect to Maurolicinae, with the subfamilies containing *Argyropelecus*, *Polyipnus*, and *Sternoptyx*; and *Argyripnus*, *Danaphos*, *Maurolicus*, and *Valenciennellus*, respectively. This further discourages the use of the historical subfamilial nomenclature. Accurate intergeneric relations within this family remain inconsistent and requires extensive analyses on the lower-level relationships. Across many studies, including herein, *Polyipnus* groups with the maurolicines rather than the other sternoptychines, refuting the accepted notion that the three deep-bodied genera form a monophyletic clade. Moreover, our study lacks representation for three maurolicine genera (*Araiophos*, *Sonoda*, and *Thorophos*), rendering a paraphyletic Maurolicinae to still be plausible. Due to the lack of taxonomic representation, we recommend provisional recognition of a 10-genus Sternoptychidae with no subfamily-level taxa.

**English name.** Hatchetfishes (*Argyropelecus*, *Polyipnus, Sternoptyx*); pearlsides (*Araiophos*, *Argyripnus*, *Maurolicus*, *Sonoda*, *Thorophos*); bottlelights (*Danaphos*); and constellationfishes (*Valenciennellus*).

**Family Stomiidae** Bleeker 1859

**Type genus:** *Stomias* Jordan & Seale 1906

**Diversity.** 327 species in 27 genera.

**Diagnosis.** Stomiidae is unique among stomiiform families in the presence of an ossified Baudelot’s ligament on the first two vertebral parapophyses; absence of gill rakers in adults; absence of the sixth hypural; division of the genyohyoideus into dorsal and ventral portions; and anterior and posterior enlargement of the posterior pelvic plate, with a cartilaginous core extending posteriorly beyond plate ossification (Additional file 4: Table S2).

**Genera included.** *Aristostomias* (6 spp.), *Astronesthes* (50 spp.), *Bathophilus* (20 spp.), *Borostomias* (6 spp.), *Chauliodus* (9 spp.), *Chirostomias* (1 sp.), *Echiostoma* (1 sp.), *Eupogonesthes* (1 sp.), *Eustomias* (134 spp.), *Flagellostomias* (1 sp.), *Grammatostomias* (4 spp.), *Heterophotus* (1 sp.), *Idiacanthus* (3 spp.), *Leptostomias* (12 spp.), *Malacosteus* (2 spp.), *Melanostomias* (18 spp.), *Neonesthes* (2 spp.), *Odontostomias* (2 spp.), *Opostomias* (2 spp.), *Pachystomias* (1 sp.), *Photonectes* (29 spp.), *Photostomias* (6 spp.), *Rhadinesthes* (1 sp.), *Stomias* (12 spp.), *Tactostoma* (1 sp.), *Thysanactis* (1 sp.), and *Trigonolampa* (1 sp.).

**Comments.** The historical delimitations of family Stomiidae have been largely uncontroversial for forty years, since the subsumption of Astronesthidae, Chauliodontidae, Idiacanthidae, Malacosteidae, Melanostomiidae, and Stomiidae into a single family in (16). Since then, these former families have retained recognition as six subfamilies within the highly-diverse but morphologically-distinct family Stomiidae (8,9,30). Other subfamilies have been proposed historically — namely Bathophilinae, Eustomiinae, Neonesthinae, and Photonectinae (3) — but have failed to gain traction, and the six traditional subfamilies of (16) have remained in common usage as recently as 2025.

Molecular investigations of Stomiid relations have frequently failed to resolve the family as monophyletic, let alone its subfamilies. Maximum-likelihood analyses using either nuclear and mitochondrial markers have either resolved the subfamily Chauliodontinae as sister to the non-stomiid genus *Ichthyococcus* (8), or to a poorly-resolved gonostomatids, phosichthyids, and sternoptychids grouping (30). However, more recent extensive phylogenetic studies have consistently resolved the traditional Stomiidae as monophyletic, including Chaliodontines within the family (9,13). Our study also resolves a monophyletic Stomiidae with *Chauliodus* as an early-branching stomiid lineage sister to *Stomias*, and as such, we acknowledge Stomiidae as a monophyletic clade, however, disregard the use of the sub-family system.

Within Stomiidae, however, no phylogenetic analysis has recovered molecular support for the prevailing subfamily system. Astronesthinae appears as either three (30) or four separate lineage (9). Malacosteinae has either been monophyletic (30), or seen to resolve as two (8) or three (9) separate lineages. Melanostomiinae has resolved in either five (9), six (30), or ten (8) separate lineages. The remaining three subfamilies — Chauliodontinae, Idiacanthinae, and Stomiinae — reliably appear as monophyletic by monotypic virtue. Our study finds unambiguous polyphylies in Astronesthinae, Malacosteinae, and Melanostomiinae, but given the overrepresentation of taxa based on a single mitochondrial marker, we advocate for a more thorough assessment using increasing molecular data. This study recognizes a monophyletic Stomiidae, containing 27 genera and no subfamily-level taxa, until such time as internal topological consensus is approached.

**English name.** Dragonfishes. Includes also stareaters (*Astronesthes*); loosejaws (*Aristostomias*, *Grammatostomias*, *Malacosteus*, *Photostomias*); boafishes (*Stomias*); snaggletooths (*Borostomias*, *Eupogonesthes*, *Heterophotus*, *Neonesthes*, *Rhadinesthes*); viperfishes (*Chauliodus*); and sawtails (*Idiacanthus*).

**Family Vinciguerriidae fam. nov.**

**Type genus:** *Vinciguerria* Jordan & Evermann 1896

**Diversity.** 6 species in 2 genera.

**Diagnosis.** Vinciguerriidae is unique among stomiiform families in the presence of an elongate hyomandibular spine, bound to the surface of the mesopterygoid by a ligament (vs short and detached from mesopterygoid in all other stomiiforms); fusion or tight adherence of the second basibranchial tooth plates (vs absent or loosely-adhering in other stomiiforms); and close adherence of the third basibranchial tooth plates on the dorsal surface (vs lateral to basibranchial).

Vinciguerriidae is further defined by a unique combination of traits. It differs from the other early-branching families (Diplophidae, Gonostomatidae, and Sternoptychidae) by: presence of a posterior photophore; gamma-type photophores; anteriorly angled dorsal uncinate process of the second pharyngobranchial (vs straight and vertical in aforementioned families); and presence of a serial photophore duct and lumen. Vinciguerriidae differs from Yarrellidae in presence of a posterior photophore; radiating A cell configuration (vs irregular in yarrellids); separate contralateral and ipsilateral branches of the premaxillary-rostrodermethmoid ligament (vs fused in yarrellids); and presence of toothplates on the fourth basibranchial. Finally, Vinciguerriidae differs from the remaining families (Ichthyococcidae, Phosichthyidae, and Stomiidae) in possessing: an anal fin originating below or anterior the dorsal fin origin (vs posterior in aforementioned families); posterior four branchiostegals separated from one another; an anterior palatomaxillary ligament looped over the dorsal surface of the lateral process of the rostrodermethmoid; a well-developed endopterygoid; medial concavity of the ascending process of the premaxillary symphysis; and presence of toothplates on the fourth basibranchial (Additional file 4: Table S2).

**Genera included.** *Pollichthys* (1 sp.) and *Vinciguerria* (5 spp.).

**Comments.** *Vinciguerria* was initially described as a diminutive “maurolicid” (33). Approximately 63 years later, the genus *Pollichthys* was similarly described and placed within Gonostomatidae at the same time as *Vinciguerria* (34). These two genera, alongside five others, were then later recognized as part of the family Phosichthyidae (28). However, initial attempts at reconciling phosichthyid interrelationships using morphology brought greater uncertainty and an inaccurate assessment of this clade’s relation to Stomiidae.

Subsequent molecular studies have largely recovered “Phosichthyidae” as paraphyletic in the manner anticipated by Harold & Weitzman (15), with the exception of the dwarf genera *Pollichthys* and *Vinciguerria*. These diminutive species reliably comprise some of the earliest-branching stomiiform genera, and in many topologies (8,30) form the sister taxon to all stomiiforms. Other analyses (9,13) resolve *Vinciguerria* and/or *Pollichthys* as separate from other “phosichthyids,” but not sister to all other stomiiforms. Our study corroborates the former outcome, with a monotypic clade containing several *Vinciguerria* species sistering to all other stomiiforms. Due to a lack of appropriate molecular data, *Pollichthys* was not included herein, but morphological (15) and molecular (30) analyses strongly indicate a sister relationship between the two genera, which we provisionally recognize even in the absence of representation.

Morphological and molecular analyses of stomiiform phylogenetics differ sharply in their recommended placement of *Vinciguerria* and *Pollichthys*. Osteologically, these genera share many characteristics with the phosichthyid-stomiid clade, hence their initial placement in Weitzman’s “Phosichthyidae”, but molecular evidence consistently dictates that they are unrelated within the order. Indeed, the dwarf genera are typified by a strange mixture of “phosichthyid” characteristics, and characteristics of the early-branching diplophids, gonostomatids, and sternoptychids. Given their unique combination of traits, and their reliable placement outside of any named family across multiple morphological and molecular studies, we recommend the recognition of a new family, Vinciguerriidae, to house these two morphologically-distinct dwarf genera until subsequent studies can resolve a stable clade containing one or both.

**English name.** Lighthousefishes (*Vinciguerria*) and stareyes (*Pollichthys*).

**Family Yarrellidae fam. nov.**

**Type genus:** *Yarrella* Goode & Bean 1896

**Diversity.** 8 species in 2 genera.

**Diagnosis.** Yarrellidae is unique among stomiiform families in the fusion of contralateral and ipsilateral branches of the premaxillary-rostrodermethmoid ligament into a continuous sheet of connective tissue (vs non-fused in all other stomiiforms). It is further defined by a unique combination of characters. Yarrellidae differs from Diplophidae, Gonostomatidae, and Sternoptychidae by the presence of a second epural (vs absence in the aforementioned families); and gamma-type photophores (vs alpha or beta). Yarrellidae is distinguishable from the remaining families (Ichthyhococcidae, Phosichthyidae, Stomiidae, and Vinciguerriidae) by: absence of a posterior orbital / postorbital photophore (vs presence in ichthyococcids, phosichthyids, stomiids, and vinciguerriids); and irregular configuration of photophore A cells (vs regular) (Additional file 4: Table S2).

**Genera included.** *Polymetme* (6 spp.) and *Yarrella* (2 spp.).

**Comments.** The genera *Yarrella* and *Polymetme* were described in 1896 and 1926, respectively, to accommodate new species that were initially identified as gonostomids. (28) shifted both genera into his “Phosichthyidae,” where they were identified as early-branching and potential outliers with respect to the rest of the family (15). This assessment was established primarily using the placement and cellular structure of light organs, which in *Polymetme* and *Yarrella* are more akin to those of gonostomatids and sternoptychids than of other phosichthyids and stomiids. Morphological analysis ultimately found *Polymetme* to form a clade with other “phosichthyids” and Stomiidae, which was in turn sister to *Yarrella*.

Recent molecular studies, including herein, have almost invariably corroborated the relationship of *Polymetme* + *Yarrella* as a unique clade that is sister to group containing *Ichthyococcus*-*Phosichthys*-*Woodsia* and the family Stomiidae (8,13,30,31). Based on previous molecular analyses and the robustness of this topology seen herein, we recommend the recognition of a new family, Yarrellidae, containing both *Polymetme* and *Yarrella*.

**English name.** Rendezvousfishes.

**Fig. S1.** All maximum-likelihood (IQ-TREE) and coalescent (ASTRAL-IV) species trees estimated in this study. Families are color-coded based on the newly proposed classification (Vinciguerriidae, Diplophidae, Stomiidae, Gonostomatidae, Sternoptychidae, Phosichthyidae, Chauliodontidae, Ichthyococcidae, outgroup) and the recently proposed classification by Smith et al. (2024) (Gonostomatidae, Sternoptychidae, Stomiidae, outgroup). (a) IQ-TREE maximum-likelihood trees (1-8) from this study, (b) ASTRAL-IV (9-14), and w-ASTRAL (15) coalescent trees from this study are colored based on our proposed classification. (c-d) IQ-TREE (16-23), ASTRAL-IV (24-29), and w-ASTRAL (30) trees colored using Smith et al. (2024) classification.


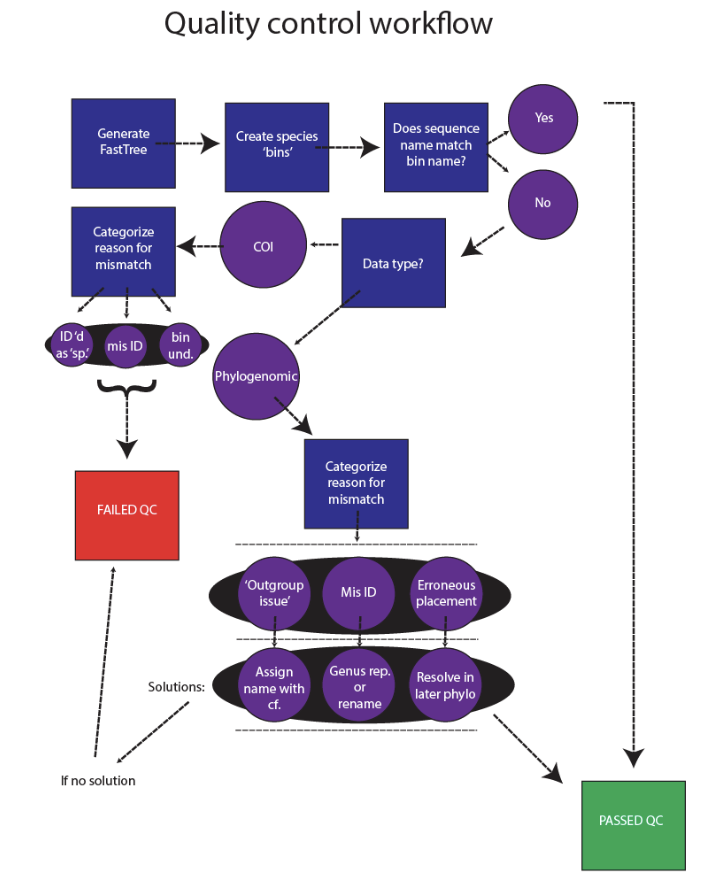


**Fig. S2.** Sequence diagram outlining the procedure undertaken to verify the quality of the acquired COI sequences from the BOLD database.

**Fig. S3.** Bar plot depicting the proportions of sequences that either passed or failed our quality control workflow and categorized by their traditional family classification.

**Fig. S4.** Morphological character matrix for the proposed stomiiform classification, based on traits defined by Harold & Weitzman (1996). Gray dashed box indicates intra-familial variation for specific traits, with numbers corresponding to the trait definitions in Harold & Weitzman (1996).

**Fig. S5.** Character optimization reconstruction mapping 51 morphological traits from Harold & Weitzman (1996) onto a cladogram representing the main phylogeny of all extant Stomiiform genera. Using parsimony, this analysis yields two equally probable hypotheses based on ACCTRAN and DELTRAN.
