# Supplementary material for: Genome-wide phylogeny reshapes our understanding of the evolution of deep-sea dragonfishes, bristlemouths, viperfishes, and allies (Stomiiformes)
Source: BMC Ecol Evol. 2025 Oct 21;25:111. doi: 10.1186/s12862-025-02453-0 (PMC12542184; doi:10.1186/s12862-025-02453-0)
Supplement: Supplementary file 2 — Additional file 2. Maximum likelihood tree using FastTree containing 2525 individuals and used for sequence quality assessment. 2474 represented solely by COI data and 51 genome-wide sequences (colored red). Clades were placed into ‘bins’ with bin names representative of a species name. 181 bins were procured, and COI sequences were colored based on families. COI data was acquired from BOLD (https://v3.boldsystems.org/). [file 12862_2025_2453_MOESM2_ESM.pdf]

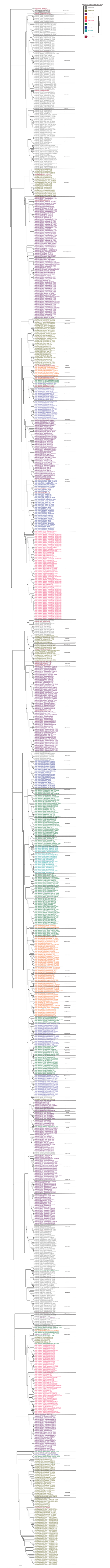

Fig. S1: All maximum-likelihood (IQ-TREE) and coalescent (ASTRAL-IV) species trees estimated in this study. Families are color-coded based on the newly proposed classification (Vinciguerriidae, Diplophidae, Stomiidae, Gonostomatidae, Sternoptychidae, Phosichthyidae, Chauliodontidae, Ichthyococcidae, outgroup) and the recently proposed classification by Smith et al. (2024) (Gonostomatidae, Sternoptychidae, Stomiidae, outgroup). (a) IQ-TREE maximum-likelihood trees (1-8) from this study, (b) ASTRAL-IV (9-14), and w-ASTRAL (15) coalescent trees from this study are colored based on our proposed classification. (c-d) IQ-TREE (16-23), ASTRAL-IV (24-29), and w-ASTRAL (30) trees colored using Smith et al. (2024) classification.
